# Supplementary material for: Volume and porosity thermal regulation in lipid mesophases by coupling mobile ligands to soft membranes
Source: Nat Commun. 2015 Jan 7;6:5948. doi: 10.1038/ncomms6948 (PMC4354032; doi:10.1038/ncomms6948)
Supplement: Supplementary Figures, Methods and References — Supplementary Figures 1-12, Supplementary Methods and Supplementary References. [file ncomms6948-s1.pdf]

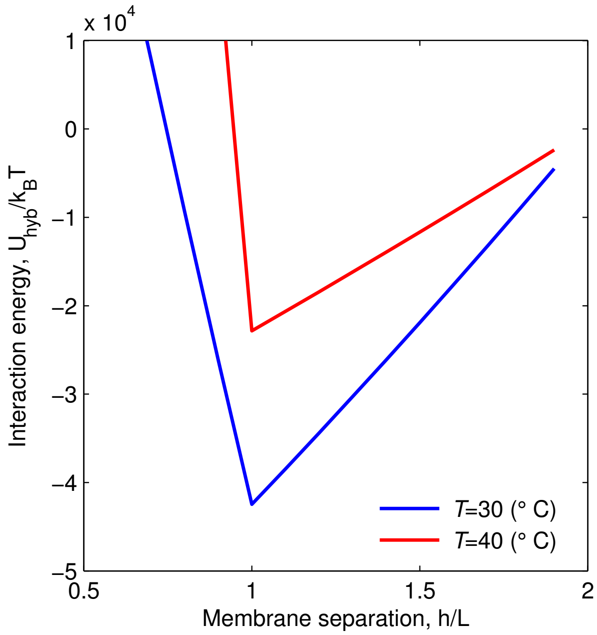

Supplementary Fig. 1. **Intraction free energy between two DNA-GUVs as a function of the membrane separation**. Note the sharp minimum at h/L=1. The geometry of the GUVs and DNA coverage are those of a typical experimental system: A = 700 μm^2, A_p = 100 μm^2, N=180000, $\Delta H^0 = -68.5$ kcal mol^{-1}, $\Delta S^0 = -193.5$ cal^{-1} mol^{-1} K^{-1}, L = 14.5 nm. Points on the horizontal axis are spaced by 0.1, the discontinuous derivative in the minimum is due to the coarse-grained description of the rigid spacer.

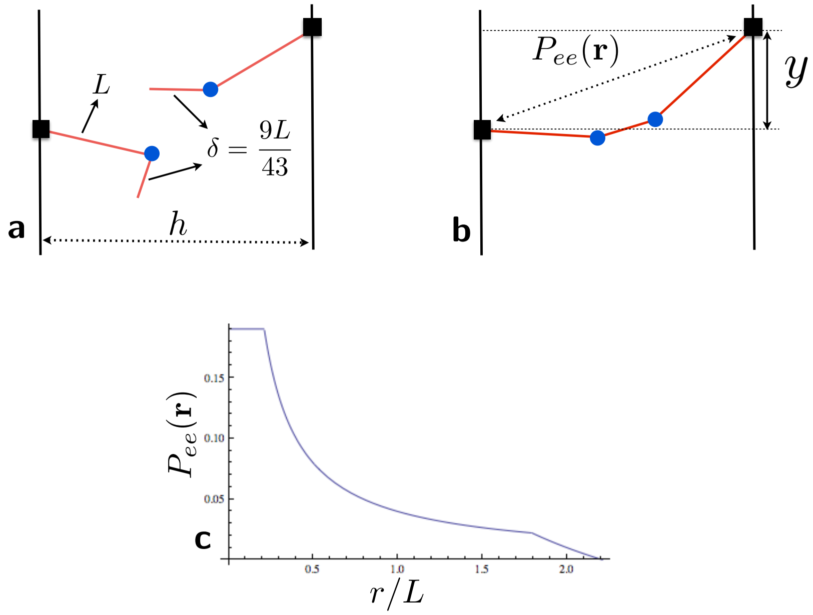

Supplementary Fig. 2. **Effect of finite-size sticky ends.** **a** A coarse grained model for free tethers that accounts for the length of the sticky ends δ. **b** Hybridised constructs are modelled as a three segment chains with the middle segment representing the dsDNA that is formed following the hybridisation of the sticky ends (for simplicity the length of this segment is also taken equal to δ). **c** End-to-end distribution function of the hybridised construct.

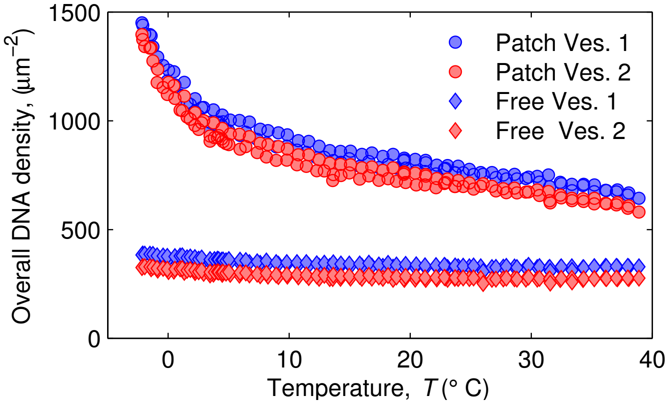

Supplementary Fig. 3. **Experimental DNA density** evaluated within ($\rho^{\rm in}_{\rm DNA} = 2N\left[\frac{x_{\rm b}}{A_{\rm p}} + \frac{1-x_{\rm b}}{A}\right]$) and outside ($\rho^{\rm out}_{\rm DNA} = 2N\frac{1-x_{\rm b}}{A}$) the patch.

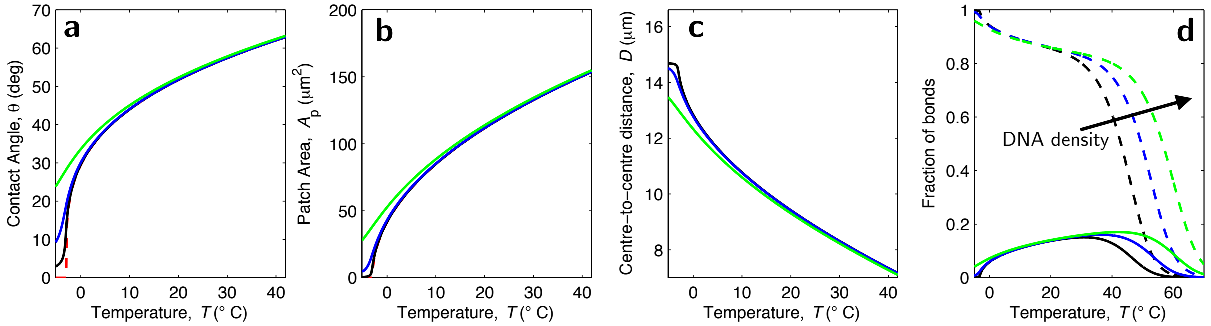

Supplementary Fig. 4. **Effect of increasing DNA concentration.** Theoretical predictions for **a** Contact angle θ, **b** patch area A_{p}, **c** bond distance D, and **d** fraction of hybridised stands at increasing DNA concentrations: $\rho_{\mathrm{DNA}} = 5 \times 10^2\ \mu\mathrm{m}^{-2}$ (black), $5 \times 10^3\ \mu\mathrm{m}^{-2}$ (blue), $5 \times 10^4\ \mu\mathrm{m}^{-2}$ (green). Red dashed lines in panels **a** and **b** indicate the zero stretching contact angle $\tilde{\theta}$ and adhesion patch area $\tilde{A}_{\mathrm{p}}$. Stronger adhesion, due to the higher DNA surface coverage ρ_{DNA}, causes the equilibrium contact angle and adhesion patch area to deviate further from the zero-stretching values. In panel **d** the solid lines indicate the fraction of bridges x_{b} and the dashed lines the fraction of loops x_{l}. As expected the temperature of the melting transition increases with ρ_{DNA}. Note that, consistently with the observations presented in the main text, larges changes in ρ_{DNA} have little influence in x_{l} and x_{b} at low temperature. Note also that in experiments we are unable to probe temperatures higher than 40°C due to the possible destabilisation of the double stranded DNA making up the inert spacers.

Model parameters: $R_0 = 7.35\ \mu\mathrm{m}$, $K_{\mathrm{a}} = 0.24\ \mathrm{N\ m}^{-1}$, $\alpha = 1.3 \times 10^{-3}\ \mathrm{K}^{-1}$, $\Delta H^0 = -68.5\ \mathrm{kcal\ mol}^{-1}$, $\Delta S^0 = -193.5\ \mathrm{cal\ mol}^{-1}\ \mathrm{K}^{-1}$, $L = 14.5$ nm, $T_0 = -2$ °C.

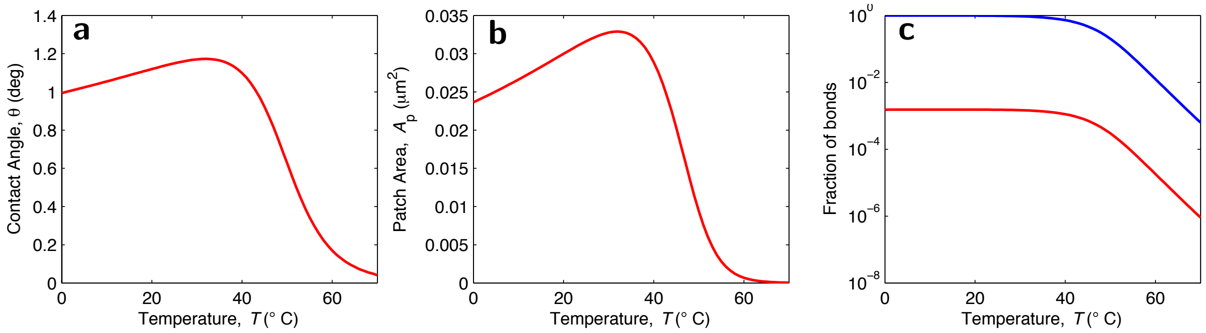

Supplementary Fig. 5. **Temperature effect on the DNA-mediated adhesion between oil droplets.** Theoretical prediction for the temperature-dependence of **a** the contact angle (θ), **b** the adhesion patch area ($A_{\rm p}$), and **c** the fraction of DNA-tethers involved in loops (blue) and bridges (red). Model parameters: $R_0 = 5$ μm, $\Delta H^0 = -68.5$ kcal mol^{-1}, $\Delta S^0 = -193.5$ cal mol^{-1} K^{-1}, $L = 14.5$ nm, $\gamma_0 = 12.5$ mN m^{-1}, $\partial\gamma_{\rm T} = -0.1$ mN m^{-1} K^{-1}, $\rho_{\rm DNA} = 500$ μm^{-2}.

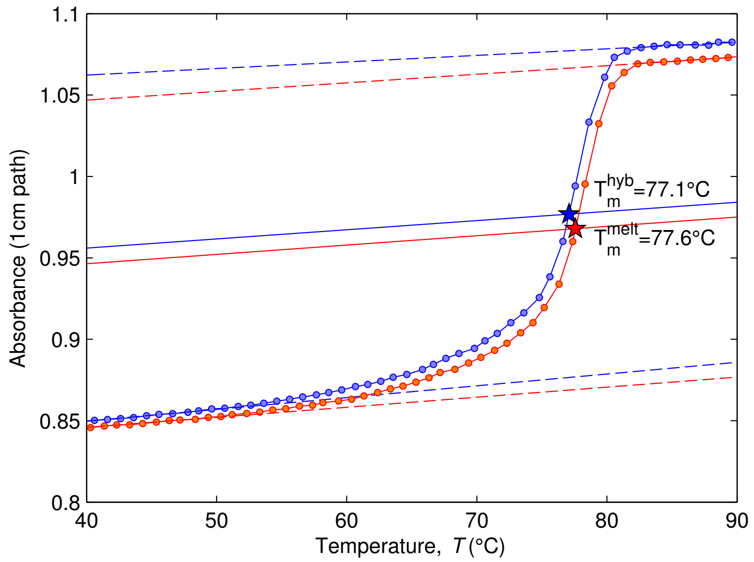

Supplementary Fig. 6. **UV-absorbance spectroscopy on assembling DNA-constructs.** Temperature dependent absorbance of a solution containing equal amounts of strands i and ii (overall 2.1 μM, see Methods section for the sequences) in TE buffer with 100 mM NaCl. Blue and red symbols are measured on cooling (hybridisation) and heating (melting) respectively. Cooling/heating rates are $\pm0.2°$C/min. Dashed lines are linear fits of the high-T ($T > 80°$C) and the low-T plateaus ($T < 50°$C). Solid lines indicate the median of the two fits. Stars indicate the melting temperatures calculates as the intersection between the absorbance curves and the solid lines. A blank solution (TE + 100mM NaCl) is used as a reference. The samples are enclosed in quartz cuvettes with 1 cm optical path (Hellma).

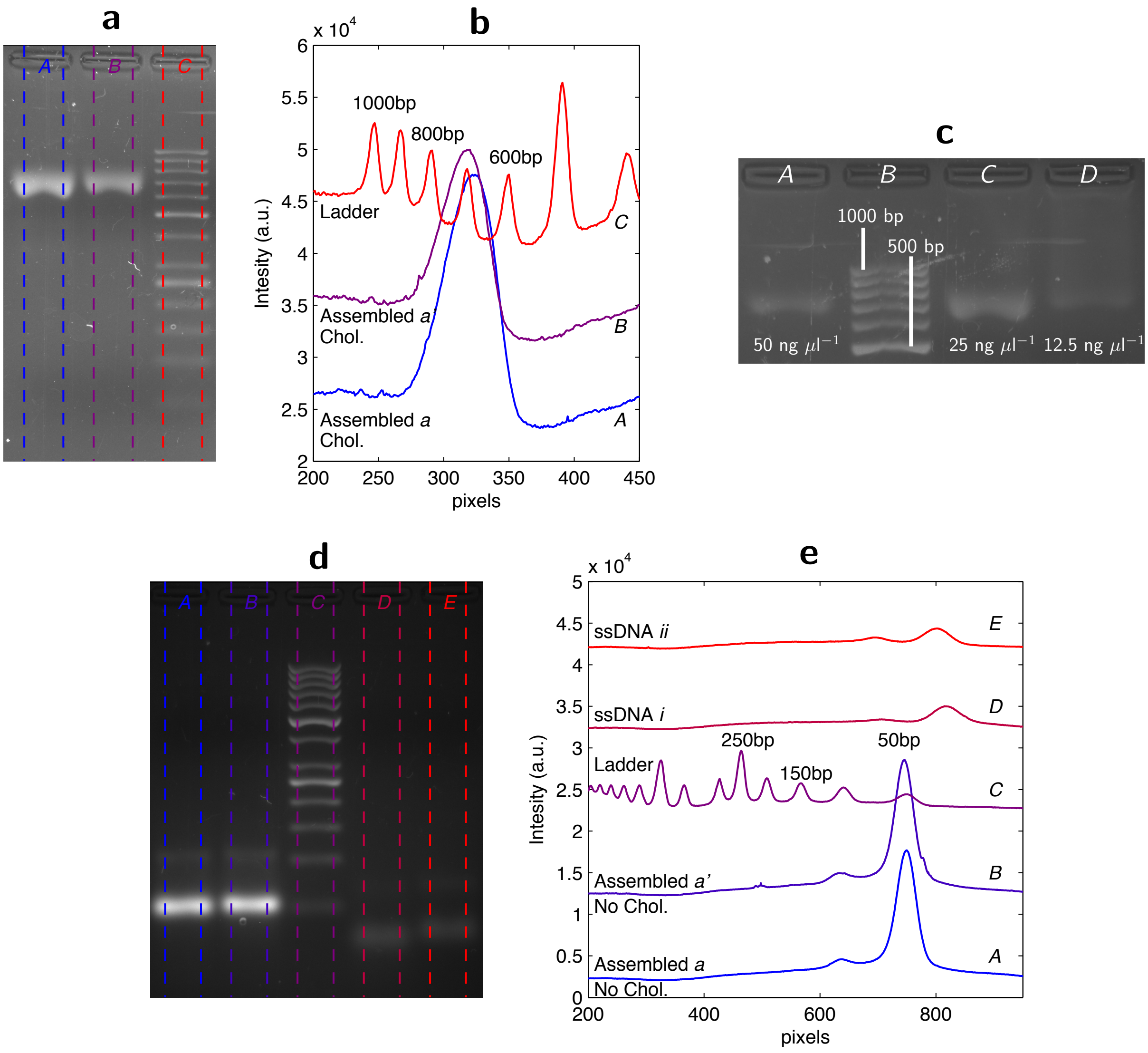

Supplementary Fig. 7. **Gel electrophoresis on assembled DNA linkers.** In panel **a** we show the fluorescence image of a gel in which lanes A and B are loaded with hyrdophobically-modified a and a' assembled constructs. Lane C contains a DNA ladder. Each lane is loaded with 20 μl of DNA solution with an overall concentration of ≈ 15 ng μl^{-1}. In panel **b** we show the fluorescence intensity of lanes A-C of panel **a**, calculated by integrating the image within the regions delimited by dashed lines (along the direction normal to the applied field). In panel **c** we show data for hyrdophobically-modified DNA at decreasing concentration (lanes A, C, and D). Lane B contains a DNA ladder. In panel **d** we show a gel in which lanes A and B are loaded with cholesterol-free a and a' assembled constructs, lane C with the ladder, and lanes D and E with ssDNA i and (cholesterol-free) ii. Each lane is loaded with 20 μl of DNA solution with an overall concentration of ≈ 80 ng μl^{-1}. **e** Intensity profiles of the 5 lanes in panel **d**.

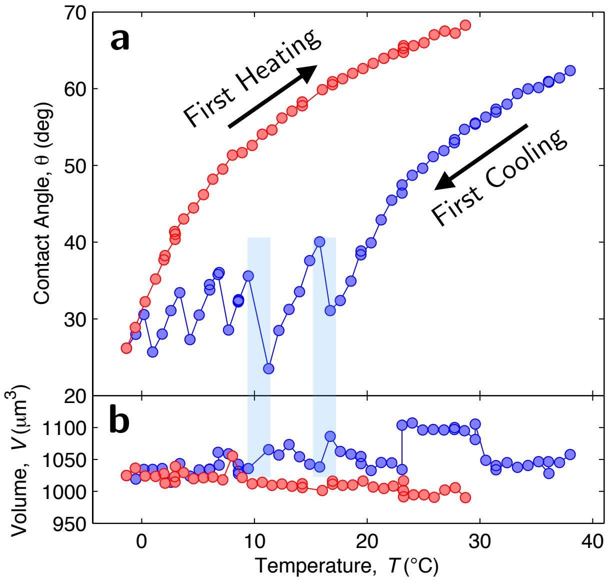

Supplementary Fig. 8. **Relaxation of the excess area. a** Temperature dependence of the contact angle of a GUVs in a pair undergoing a cooling/heating cycle for the first time. **b** Volume of the same liposome. Blue symbols refer to the cooling ramp, red symbols to the heating ramp. Light blue regions highlight the correspondence between sudden increases in θ and sudden drops in V.

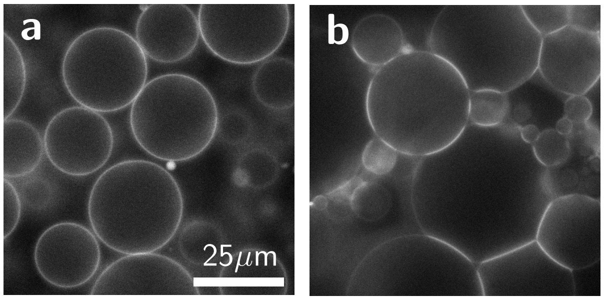

Supplementary Fig. 9. **Checking for the specificity of the binding**. Snapshot of a sample with vesicles coated with a' DNA tethers only (**a**) , and both a and a' tethers (**b**). Both images are taken at room temperature.

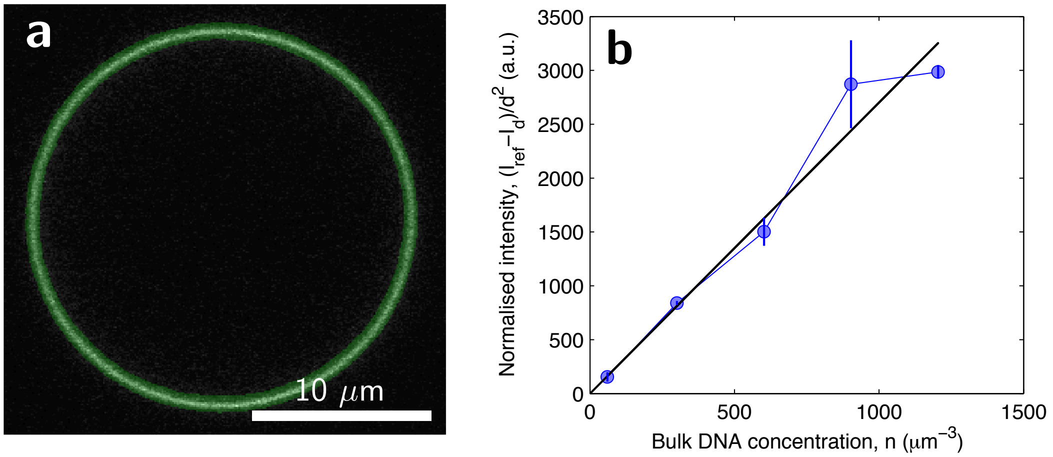

Supplementary Fig. 10. **Quantifying the coating density**. **a** Confocal image of a single GUV used for estimating the DNA coverage density. The green shaded area highlights the region used to evaluate the average fluorescent intensity. **b** Fluorescent intensity of the reference DNA samples, diminished by the dark intensity and normalised by the pixel area, as a function of the bulk DNA concentration (symbols). The solid line is a linear fit used to extract C.

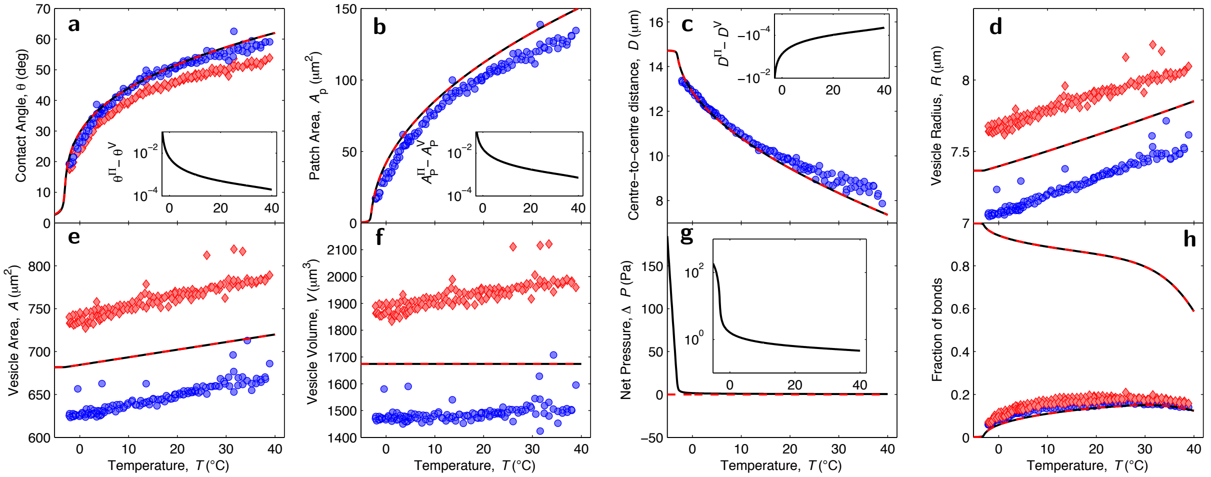

Supplementary Fig. 11. **Constant volume and osmotically equilibrated vesicles**. **a** Equilibrium contact angle θ, **b** adhesion patch area A_{p}, **c** bond distance D, **d** vesicle radius R, **e** vesicle total area A, and **f** vesicle volume V. In panel **g** we show the theoretical calculation for the net pressure across the membranes $\Delta P = P - \Pi$, where P is the Laplace pressure defined in Supplementary Equation 2 and Π is the osmotic pressure drop defined in Supplementary Equation 1. In the inset we show the same graph in semi-log scale. In panels **a-g**, symbols indicate experimental data, black solid lines indicate theoretical predictions calculated at constant volume, red dashed lines indicate theoretical predictions calculated at zero pressure. In the insets of panels **a-c**, we highlight the difference between the estimates computed with the assumptions of constant volume (V) and the osmotically equilibrated (Π) GUVs. **h** Theoretical prediction for the fraction of tethers involved in bridges (x_{b}, solid line) and loops (x_{l}, dashed line). Symbols indicate experimental estimates of x_{b}. Model parameters: $K_{\mathrm{a}} = 0.24$ N m^{-1}, $\alpha = 1.3 \times 10^{-3}$ K^{-1}, $\rho_{\mathrm{DNA}} = 390$ μm^{-1}, $\Delta H^0 = -68.5$ kcal mol $^{-1}$, $\Delta S^0 = -193.5$ cal mol^{-1} K^{-1}, $L = 14.5$ nm, $\rho_{\mathrm{in}} = \rho_{\mathrm{out}} = 300$ mM. Fitting parameter $T_0 = -3$ °C.

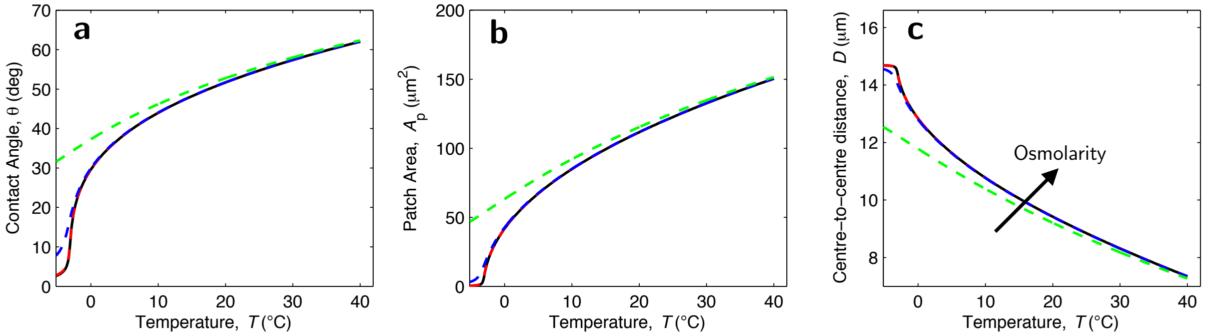

Supplementary Fig. 12. **Osmotically-equilibrated vesicles at lower solute concentrations**. **a** Equilibrium contact angle θ, **b** adhesion patch area A_p, **c** centre-to-centre distance D. Black solid lines indicate theoretical predictions calculated at constant volume, dashed lines indicate theoretical predictions calculated at constant osmotic pressure and decreasing osmolarity $\rho_\mathrm{in} = \rho_\mathrm{out} = 300$ mM (red), 10 mM (blue) 1 mM (green). Model parameters: $R_0 = 7.35\ \mu$m, $K_\mathrm{a} = 0.24$ N m^{-1}, $\alpha = 1.3 \times 10^{-3}$ K^{-1}, $\rho_\mathrm{DNA} = 390\ \mu$m^{-2}, $\Delta H^0 = -68.5$ kcal mol^{-1}, $\Delta S^0 = -193.5$ cal mol^{-1} K^{-1}, $L = 14.5$ nm, $T_0 = -2$ °C.

SUPPLEMENTARY METHODS

Electroformation

GUVs are prepared by electroformation [1, 2]. DOPC is purchased in chloroform (25 mg/ml) from Avanti Polar Lipids (Alabaster, AL) and stored at -20°C. The stock lipid solution is diluted to 3.57 mg/ml and a volume of 160 μl is spin-coated onto a Indium Tin Oxide (ITO) coated microscope slide (Visiontek Systems Ltd.) at 600 rpm for 2 min [3]. The lipid-coated slide is then left to dry under vacuum for at least 1 hour. Together with a second clean ITO slide, the lipid-coated slide is arranged to form a capacitor cell where the two conductive surfaces are facing inwards, and are separated by a U-shaped silicone rubber spacer (thickness 0.5 mm, Altec Products Limited). The chamber is filled with a degassed and filtered (0.22 μm syringe filter) 300mM sucrose (Sigma Aldrich) solution in double-distilled water (18.2 MΩcm resistance). The filled chamber is then sealed and connected to a function generator. For the formation, a sinusoidal potential with 1 V amplitude (peak-to-peak) is applied with a frequency of 10 Hz for 2 hours and of 2 Hz for 1 hour. Because of the low melting temperature of DOPC (-17°C) the process can be carried out at room temperature. After formation the vesicles are collected from the chamber using a pipette, stored in an Eppendorf tube at room temperature and used within 3-4 days.

UV absorbance spectroscopy

The hybridisation protocol used to assemble the DNA constructs from the two single-stranded DNA sequences *i* and *ii* (see Methods section) is tested by monitoring absorbance at 260 nm on a UV-visible absorbance spectrometer (Varian Cary 300 Bio). A sample is hybridised (cooled down) and melted (heated up) again at the same rate used on the PCR machine ($\pm 0.2°$C min^{-1}). As shown in Supplementary Fig. 6 the hybridisation and melting curves almost overlap, indicating an equilibrium self-assembly of the constructs. We fit the high and low-temperature plateaus of the hybridisation/melting profiles with straight lines (baselines), then measure the melting temperature of the constructs as the intersection between the median of the two baselines and the absorbance curve (linearly interpolated) [4]. The melting temperatures measured on cooling and heating are compatible within the experimental error [4].

Gel electrophoresis

The correct assembly of the constructs is further checked using gel electrophoresis. Briefly, we prepare 100 ml of 3% agarose (Sigma Aldrich) gel in 1× TBE buffer (Tris-Borate-EDTA, prepared from 10× concentrate solution, Sigma Aldrich) and 10 μl SYBR Safe DNA Gel Stain (Life Technologies). The samples are loaded using blue loading buffer (Life Technologies). Electrophoresis is performed in a horizontal tray with 60V applied voltage for 160 minutes. As a reference, one of the lanes is loaded with a DNA ladder (Generuler 50-1000 bp, Life Technologies). In Supplementary Fig. 7a we show a gel in which lanes A and B contain assembled linkers carrying a and a' sticky ends, lane C is loaded with the ladder. The quantitative analysis of the fluorescent intensity showed in panel Supplementary Fig. 7b demonstrates that the assembled linkers tend to aggregate into superstructures with high molecular weight, corresponding to 800-600 bp on the ladder. This is expected since hydrophobically modified DNA can assemble into micelles and liposome-like structures [5]. In Supplementary Fig. 7c, we repeat the test at decreasing cholesterol-DNA concentration in order to identify the critical micellar concentration (CMC) of the superstructures. When decreasing the concentration at 12.5 ng μl^{-1} the relatively sharp band observed at higher concentrations is replaced by a smeared track caused by the non-specific adhesion of the amphiphiles on the gel, caused in turn by the presence of exposed cholesterols. From this we can argue that the CMC is $\gtrsim$12.5 ng μl^{-1}. In our coating procedure we use an overall DNA concentration of 0.16 ng μl^{-1}, safely below this value. Therefore we expect the micelles not to play a role in the coating procedure nor in the DNA-mediated interaction between the GUVs.

To verify that the superstructures are indeed due to the hydrophobic modification, we repeat the electrophoresis with a and a' linkers assembled from cholesterol-free ii strands (see Methods). As demonstrated in Supplementary Fig. 7d-e, the cholesterol-free linkers line up in correspondence of the 50 bp band of the ruler, indicating a correct assembly. The faint secondary peak appears around 100 bp, indicating a weak non-specific pairing. Lanes D and E are loaded with single-stranded i and (cholesterol-free) ii DNA. As expected, these exhibit broad peaks in the low molecular weight (< 50) bp region of the gel.

Osmotically equilibrated vesicles

In this section we compute the relation between R and $\hat{\theta}$ (analogue to Eq. 26, main text) in the limit of water-permeable vesicles. This can be done by imposing a balance between the osmotic pressure across the membrane and the Laplace pressure [6]. The former is given by

$$\Pi = k_{\mathrm{B}}T\left(\frac{V_0}{V}\rho_{\mathrm{in}} - \rho_{\mathrm{out}}\right), \tag{1}$$

where ρ_{in} and ρ_{out} are the solute density respectively within and outside the GUVs.
The Laplace pressure can be expressed as

$$P = \frac{2\sigma}{R}, \tag{2}$$

where the membrane tension σ is

$$\sigma = K_{\mathrm{a}}\frac{A-\tilde{A}}{\tilde{A}}. \tag{3}$$

By equating Supplementary Equations 1 and 2, and using Supplementary Equation 3, we obtain

$$\frac{R}{R_0}\left(\frac{V_0\rho_{\mathrm{in}}}{V\rho_{\mathrm{out}}} - 1\right) = \nu\left(\frac{A}{\tilde{A}} - 1\right), \tag{4}$$

where we define

$$\nu = \frac{2K_{\mathrm{a}}}{k_{\mathrm{B}}T\rho_{\mathrm{out}}R_0}. \tag{5}$$

Finally, by substituting Eqs. 23, 24, and 25 of the main text into Supplementary Equation 4 we obtain

$$\frac{R}{R_0}\left[\frac{4\rho_{\mathrm{in}}}{\rho_{\mathrm{out}}\left(1+\cos\hat{\theta}\right)^2\left(2-\cos\hat{\theta}\right)}\left(\frac{R_0}{R}\right)^3 - 1\right] = \nu\left[\frac{\left(1+\cos\hat{\theta}\right)\left(3-\cos\hat{\theta}\right)}{4\left[1+\alpha\left(T-T_0\right)\right]}\left(\frac{R}{R_0}\right)^2 - 1\right]. \tag{6}$$

By numerically solving the fourth-order equation in Supplementary Equation 6 (using a built in Matlab® function) we can extract $R(\hat{\theta})$.
In Supplementary Fig. 11 we compare experimental results with predictions calculated in the constant-volume case (Eq. 26, main text) and in the osmotically-equilibrated case (Supplementary Equation 6). No significant differences are noticed in any of the experimental observables. The two assumptions give clear differences for $\nu \gg 1$. This condition is verified for very low osmotic pressure, as shown in Supplementary Fig. 12, with the osmotically equilibrated GUVs showing significantly larger contact angles and adhesion patch areas. A similar scenario would occur for smaller liposomes ($R_0 \approx 1$ μm).

Effect of varying inter-membrane distance

In the main body of the paper the hybridisation free energy $U_{\rm hyb}$ (Eq. 10 of the main text) is derived under the assumption of fixed intermembrane distance $h = L$. Here we derive $U_{\rm hyb}$ for arbitrary $0 \le h \le 2L$ and demonstrate that $U_{\rm hyb}(h)$ displays a very strong minimum at $h = L$, which justifies the use of the simplified theory presented in the main body. We follow the same conceptual steps explained in the main body for the constrained theory. The only difference is that for $h < L$ loops and unbound tethers have different chemical potentials depending on their positions on the membrane: within or outside the patch region. This effect is due to the excluded volume interactions between DNA tethers and the two opposing flat membrane walls. Consequently, rather than only bridges and loops, here we have to consider more kinds of possible *states*, namely: bridges (b), loops compressed within the patch region, (l,c), loops outside patch region (l,nc), free tethers compressed within the patch region (f,c), and free tethers outside the patch region (f,nc).

First we generalise the hybridisation free energies. As in Eq. 6 of the main text, we define $\Delta G_{\rm X}(h) = \Delta G^0 - T\Delta S^{\rm conf}_{\rm X}(h)$ (with X=b, X=l,nc, or X=l,c). Since no hybridisation takes place, the free energy for compressed tethers contains only the entropic term $\Delta G_{\rm f,c} = -T\Delta S^{\rm conf}_{\rm f,c}$. For a fixed geometry, the confinement entropy for the specie X is obtained as (cnf. Eq. 28 of the main text after the introduction of the translational entropy term)

$$\exp[\Delta S^{\rm cnf}_{\rm X}/k_{\rm B}] = \frac{\hat{A}_{\rm X}}{A^2}\int_{d_t \le 2L} \frac{f_{\rm cut}({\rm X}; d_t, h)}{\rho_0 L^2}\frac{y\cdot {\rm d}y}{d_t}\,, \tag{7}$$

where $\hat{A}_{\rm b} = \hat{A}_{\rm l,c} = A_{\rm p}$ and $A_{\rm l,nc} = A - A_{\rm p}$. As in the Methods section, $f_{\rm cut}$ is the planar angle available to an hybridised constructs divided 2π. For the case of bridges we obtain (see also Eqs. A1-A3 of [7])

$$f_{\rm cut}({\rm b}; d_t, h) = \tag{8}$$
$$= \begin{cases} 1 - \frac{2}{\pi}\arccos\left(\tan^{-1}\left(\arccos\frac{d_t}{2L}\right)\tan^{-1}\left(\arccos\frac{h}{d_t}\right)\right) & \text{if } \arccos\left(\frac{h}{d_t}\right) > \frac{\pi}{2} - \arccos\left(\frac{d_t}{2L}\right) \\ 1 & \text{if } \arccos\left(\frac{h}{d_t}\right) \le \frac{\pi}{2} - \arccos\left(\frac{d_t}{2L}\right), \end{cases}$$

while for loops within the patch region we have

$$(f_{\rm cut}({\rm l}, c; d_t, h) = \begin{cases} \frac{1}{2} & \text{if } h \ge \sqrt{L^2 - \frac{d_t^2}{4}} \\ \frac{1}{2} - \frac{1}{\pi}\arccos\left(\frac{h}{\sqrt{L^2 - d_t^2/4}}\right) & \text{if } h < \sqrt{L^2 - \frac{d_t^2}{4}}. \end{cases} \tag{9}$$

For loops outside the patch region we simply have $f_{\rm cut} = 1/2$.
Using the expressions for $f_{\rm cut}$ we can integrate Supplementary Equation 7 and derive explicit expression for the configurational entropy terms

$$\exp[\Delta S^{\rm conf}_{\rm b}(h)/k_{\rm B}] = \frac{A_{\rm p}}{A^2}\frac{1}{\rho_0 L}\cdot\begin{cases} h & \text{if } h < L \\ 2 - h & \text{if } L < h < 2L \end{cases} \tag{10}$$

$$\exp[\Delta S^{\rm conf}_{\rm l,nc}/k_{\rm B}] = \frac{(A - A_{\rm p})}{A^2}\frac{1}{\rho_0 L}\cdot\begin{cases} h & \text{if } h < L \\ 1 & \text{if } L < h < 2L \end{cases} \tag{11}$$

$$\exp[\Delta S^{\rm conf}_{\rm l,c}/k_{\rm B}] = \frac{A_{\rm p}}{A^2}\frac{1}{\rho_0 L}. \tag{12}$$

For free tethers we obtain

$$\exp[\Delta S^{\rm conf}_{\rm f,c}/k_{\rm B}] = \frac{A_{\rm p}}{A^2}\cdot\begin{cases}\frac{h}{L} & \text{if } h<L\\ 1 & \text{if } L<h<2L\end{cases}$$
$$\exp[\Delta S^{\rm conf}_{\rm f,nc}/k_{\rm B}] = \frac{(A-A_{\rm p})}{A^2}\,. \tag{13}$$

Using Supplementary Equations 12 and 13 we can calculate $\Delta G_{\rm X}$, and derive an expression for $U_{\rm hyb}$ following the same procedure outlined in the Methods section of the constrained theory. First we write the partition function of a system featuring $N^{\rm l,c}$ compressed loops, $N^{\rm l,nc}$ non compressed loops, $N^{\rm b}$ bridges, and $N^{\rm f,c}$ compressed free strands. This is done by generalising Eqs. 29 and 30 of the main text but, for practical reasons, assuming equal number bridges and loops on the two GUVs. This is by no means a limitation in view of the fact that the solution of the self-consistent equations (Eqs. 35 and 36 of the main text) is symmetric under permutation of the two GUVs. The partition function then becomes

$$\exp[-\beta U_{\rm hyb}] = \sum \Omega(N^{\rm l,c}, N^{\rm l,nc}, N^{\rm b}, N^{\rm f,c})\exp[-2N^{\rm b}\beta\Delta G_{\rm b} - 2N^{\rm l,c}\beta\Delta G_{\rm l,c} - 2N^{\rm l,nc}\beta\Delta G_{\rm l,nc}$$
$$+4\Delta S^{\rm conf}_{\rm f,c}N^{\rm f,c}/k_B + 4\Delta S^{\rm conf}_{\rm f,nc}N^{\rm f,nc}/k_{\rm B}] \tag{14}$$
$$\Omega(N^{\rm l,c}, N^{\rm l,nc}, N^{\rm b}, N^{\rm f,c}) = \binom{N}{N^{\rm f,c}}^4\binom{N-N^{\rm f,c}}{N^{\rm l,nc}}^4(N^{\rm l,nc})!^2\binom{N-N^{\rm f,c}-N^{\rm l,nc}}{N^{\rm l,c}}^4(N^{\rm l,c})!^2$$
$$\binom{N-N^{\rm f,c}-N^{\rm l,nc}-N^{\rm l,c}}{N^{\rm b}}^4(N^{\rm b})!^2$$

Note that $N^{\rm f,nc}$ is not a free variable, since $N^{\rm f,nc} = N - N^{\rm b} - N^{\rm l,nc} - N^{\rm l,c} - N^{\rm f,c}$. We define fractions of DNA assemblies and unbound tethers as

$$x_{\rm b} = \frac{N^{\rm b}}{N} \qquad x_{\rm l,c} = \frac{N^{\rm l,c}}{N} \qquad x_{\rm l,nc} = \frac{N^{\rm l,nc}}{N} \qquad x_{\rm f,c} = \frac{N^{\rm f,c}}{N} \tag{15}$$

and include the non-extensive combinatorial term into the hybridisation free energy

$$\Delta G^*_{\rm X} = \Delta G_{\rm X} - k_{\rm B}T\log N\,. \tag{16}$$

Following the same procedure that led to Eqs. 35 36 and 10 of the main text we find the following self consistent (SC) equations for the stationary solution

$$\bar{x}_{\rm b} = (1-\bar{x}_{\rm b}-\bar{x}_{\rm l,nc}-\bar{x}_{\rm l,c}-\bar{x}_{\rm f,c})^2\exp[-\beta\Delta G^*_{\rm b}(h) - \Delta S^{\rm conf}_{\rm f,nc}/k_{\rm B}]$$
$$\bar{x}_{\rm l,nc} = (1-\bar{x}_{\rm b}-\bar{x}_{\rm l,nc}-\bar{x}_{\rm l,c}-\bar{x}_{\rm f,c})^2\exp[-\beta\Delta G^*_{\rm l,nc}(h) - \Delta S^{\rm conf}_{\rm f,nc}/k_{\rm B}]$$
$$\bar{x}_{\rm l,c} = (1-\bar{x}_{\rm b}-\bar{x}_{\rm l,nc}-\bar{x}_{\rm l,c}-\bar{x}_{\rm f,c})^2\exp[-\beta\Delta G^*_{\rm l,c}(h) - \Delta S^{\rm conf}_{\rm f,nc}/k_B]$$
$$\bar{x}_{\rm f,c} = (1-\bar{x}_{\rm b}-\bar{x}_{\rm l,nc}-\bar{x}_{\rm l,c}-\bar{x}_{\rm f,c})\exp[\Delta S^{\rm conf}_{\rm f,c}(h)/k_{\rm B} - \Delta S^{\rm conf}_{\rm f,nc}/k_{\rm B}] \tag{17}$$

It is easy to see that for $h = L$ the solution of Supplementary Equation 17 are equivalent to the solutions of Eqs. 35 and 36 of the main text. For instance if $h = L$ we have $\exp[\Delta S^{\rm conf}_{\rm f,c}(h)/k_{\rm B} - \Delta S^{\rm cnf}_{\rm f,nc}/k_{\rm B}] = A_{\rm p}/(A-A_{\rm p})$ and the last of Supplementary Equations 17 predicts that free tethers in the patch region and outside the patch region are distributed, as expected, according to $\bar{x}_{\rm f,c}/\bar{x}_{\rm f,nc} = A_{\rm p}/(A-A_{\rm p})$. Similar considerations apply for loops.

We can now calculate $U_{\rm hyb}$ by substituting the the saddle point solutions (Supplementary Equations 17) into the free energy Supplementary Equation 14 (expanded as done in Eq. 32 of the main text). We obtain the final expression

$$U_{\rm hyb} = k_{\rm B}TN[4\log(1 - \bar{x}_{\rm b} - \bar{x}_{\rm l,nc} - \bar{x}_{\rm l,c} - \bar{x}_{\rm f,c}) + 2\bar{x}_{\rm b} + 2\bar{x}_{\rm l,nc} + 2\bar{x}_{\rm l,c}] \quad (18)$$

In Supplementary Fig. 1 we plot $U_{\rm hyb}$ at different temperatures for a typical experimental system. In particular we prove that the minimum of the interaction is reached at $h = 1$, therefore we can safely use the constrained version of the theory for comparison with experimental data.
This guarantees a uniform distribution of loops and untethered DNA within and outside the patch area.

Point-like reactive spot approximation

To derive the configurational entropy term in Eq. 7 of the main text we neglect the physical size of the sticky ends and map them into point particles. Under this assumption unbound tethers are modelled as rigid rods of length L, equal to the length of the double stranded spacers (whose persistence length is $\approx 3L$) whereas bound constructs are modelled as two segments of the same length with a fully flexible connection (see Fig. 3a of the main text). In this section we quantify systematic errors following from this assumption by considering an improved model in which free constructs are made of two fully flexible segments of length L and $\delta = 9L/43$ [Supplementary Fig. 2a], and in which hybridised constructs are made of two segments of length L chained by a segment of length δ [Supplementary Fig. 2b], where δ accounts for the length of the sticky end (9 bases compared with the 43 bases of the ds spacer).

The configurational entropy can be evaluated from the partition functions (including both rotational and translational degrees of freedom) of the free (Z_{free}) and hybridised ($Z_{\mathrm{l/b,hyb}}$) constructs and is given by

$$\exp[\Delta S^{\mathrm{conf}}_{\mathrm{l/b}}/k_{\mathrm{B}}] = \frac{4\pi\delta^2}{\rho_0}\frac{Z_{l/b,\mathrm{hyb}}}{Z_{\mathrm{free}}}\,. \tag{19}$$

In Supplementary Equation 19 a pre-factor equal to $4\pi\delta^2$ has been introduced to avoid double counting the loss in rotational degrees of freedom of the sticky-ends segments that is already accounted by ΔG^0 in the hybridisation free energy of the free segments in solution. Notice that, as in the main text, we have $\Delta G_{\mathrm{l/b}} = \Delta G^0 - T\Delta S^{\mathrm{conf}}_{\mathrm{l/b}}$.

The partition function of the free tethers is given by

$$Z_{\mathrm{free}} = (4\pi^2 L\delta A)^2\left[2L\delta - \frac{\delta^2}{2}\right]^2. \tag{20}$$

In Supplementary Equation 20 and in what follows, we neglect steric interactions between DNA tethers and the opposite membranes within the patch area. This is the case if $h = L + \delta \approx L$. The partition function of the hybridised constructs is given by

$$Z_{\mathrm{l/b,hyb}} = f^{\mathrm{cut}}_{l/b}\hat{A}_{\mathrm{l/b}}(4\pi L^2)^2(4\pi\delta^2)\int_{|\mathbf{y}|<d_t}\mathrm{d}\mathbf{y}P_{ee}(d_t)\,, \tag{21}$$

where, as defined in the main text, d_t is the distance between the two tethering points, y is their lateral displacement (see Fig. 3a of the main text), and $f^{\mathrm{cut}}_{\mathrm{l}} = 1/2$ $f^{\mathrm{cut}}_{\mathrm{b}} = 1$. P_{ee} is the end-to-end distribution function of a chain made of three segments, two of length L and one of length δ, which is given by [Supplementary Fig. 2c]

$$P_{ee}(\mathbf{r}) = \frac{1}{16\pi\delta L^2}\begin{cases} 2 & \text{if } r<\delta \\ 2\frac{\delta}{r} & \text{if } \delta<r<2L-\delta \\ \frac{\delta+2L-r}{r} & \text{if } 2L-\delta<r<2L+\delta \end{cases} \tag{22}$$

Using Supplementary Equations 20 and 22 in Supplementary Equation 19 we obtain

$$\Delta G^{\mathrm{conf}}_{\mathrm{l/b}} = -k_{\mathrm{B}}T\log\left[\frac{1}{\rho_0 L}\frac{\hat{A}_{\mathrm{l/b}}}{A^2}\right] + k_{\mathrm{B}}T\chi_{l/b} \tag{23}$$

The term χ quantifies the deviation between the current generalised model and the one used in the main body (Eq. 7), and is given by

$$\chi_{\mathrm{b}} = -\log\left[4L\int \mathrm{d}\mathbf{y}\, P_{ee}(\sqrt{h^2+\mathbf{y}^2})(1-\frac{\sigma}{4L})^{-2}\right] \tag{24}$$

$$\chi_{\mathrm{l}} = -\log\left[2L\int \mathrm{d}\mathbf{y}\, P_{ee}(y)(1-\frac{\sigma}{4L})^{-2}\right] \tag{25}$$

When $h = L$ we find $\chi_{\mathrm{b}} = 0.11\, k_B T$ and $\chi_{\mathrm{l}} = 0.055\, k_{\mathrm{B}} T$ meaning that the influence of the length of the sticky end is negligible when compared, for instance, with inert tails corrections [8].

Energy of the reference state

Here we estimate the reference energy U_0 in Eq. 5 of the main text, encoding for the energy of a pair of isolated vesicles

$$U_0 = U_0^{\text{stretching}} + U_0^{\text{DNA}}. \tag{26}$$

The stretching contribution is only present for pre-stretched vesicles, i.e. if the reduced volume is $v > 1$ (i.e. $T < T_0$) [6]

$$U_0^{\text{stretching}} = \begin{cases} 0 \text{ if } T \geq T_0 \\ K_{\text{a}} \frac{\left(A_0 - \tilde{A}\right)^2}{A_0} \text{ if } T < T_0. \end{cases} \tag{27}$$

The term U_0^{DNA} can be calculated as the free energy of the $2N$ DNA linkers on two isolated vesicles of total area equal to the unstretched area $\tilde{A}$, which therefore can form only loops. The hybridisation free energy for the formation of a single loops is:

$$\Delta G_{\text{l}}^{i} = \Delta G_0 - k_{\text{B}} T \log \left[\frac{1}{\rho_0 L \tilde{A}} \right]. \tag{28}$$

The fraction of tethers involved in loops is:

$$\bar{x}_{\text{l}}^{i} = \frac{1 + 2q_{\text{l}}^{i} - \sqrt{1 + 4q_{\text{l}}^{i}}}{2q_{\text{l}}^{i}}, \tag{29}$$

with

$$q_{\text{l}}^{i} = \exp\left(-\beta \Delta G_{\text{l}}^{*i}\right), \tag{30}$$

and

$$\Delta G_{\text{l}}^{*i} = \Delta G_{\text{l}}^{i} - k_{\text{B}} T \log N \tag{31}$$

The overall reference free-energy is therefore

$$U_0^{\text{DNA}} = 2N k_{\text{B}} T \left[\log\left(1 - \bar{x}_{\text{l}}\right) + \bar{x}_{\text{l}}\right]. \tag{32}$$

Note that U_0 does not depend on the contact angle $\hat{\theta}$.

DNA-mediated adhesion in emulsion droplets

In this section we extend our theory to the case of DNA-functionalised emulsion droplets. We demonstrate that the re-entrant shrinking of the adhesion patch is negligible if compared with the case of GUVs. The calculation of the free energy for the case of droplets can be done using the same equations developed for vesicles at fixed volume. In particular we can use Eqs. 23-25 (main text) to compute the radius, the area of the patch, and the total area of the droplet as a function of the contact angle $\hat{\theta}$ and the total volume as specified by R_0 ($V = 4\pi R_0^3/3$). Moreover Eq. 13 (main text) provides the DNA mediated interactions between the patches U_{DNA} with the fraction of bridges and loops (x_b and x_l) that can be computed using Eqs. 10-12(main text), paying attention to the dependence in $\hat{\theta}$ of the configurational hybridisation free energy. The key difference is that, for droplets, the stretching term given by Eq. 5 (main text) is replaced by the surface tension contribution resulting in the following free energy

$$U(\hat{\theta}) = U_{DNA}(\hat{\theta}) - U^0_{\mathrm{DNA}} + \gamma \left[A(\hat{\theta}) - A_0\right] \tag{33}$$

where $A_0 = 4\pi R_0^2$ and U^0_{DNA} is given by Supplementary Equation 32. The temperature dependence of the interfacial tension γ can be parametrised as $\gamma = \gamma_0 + \partial\gamma_\mathrm{T}\,(T - 273.15\mathrm{K})$, with $\partial\gamma_\mathrm{T} < 0$ [9]. For the typical case of silicone oil droplets in water we have $\gamma_0 \approx 12.5$ mN m^{-1} [10]. The parameter $\partial\gamma_\mathrm{T}$ is in the range of -0.1 mN m^{-1} K^{-1} for the most of the commonly used oils [9]. In Supplementary Fig. 5a-b we report the temperature-dependence of the contact angle and the patch size obtained by minimising Supplementary Equation 33. Notice that differently from vesicles, the theory for droplets predicts a shrinking of the adhesion area upon heating above the melting temperature of the DNA-strands. This effect is absent in vesicles due to the zero-stretching deformation that at high temperatures enables adhesion with no energy cost. The slight decrease in θ and A_p observed in droplets upon cooling is partially due to the temperature dependence of γ and partially to the $\hat{\theta}$-dependence of the configurational contribution to the hybridisation free energies. In the temperature interval $40 - 0$°C this re-entrance leads to a 20% reduction in θ, as compared with the 80% reduction observed in GUVs. In Supplementary Fig. 5c we show the temperature dependence of the fraction of tethers involved in loops and bridges. The rigidity of the droplets allows only the formation of small adhesion patches, leading to a strong entropic penalty for bridges formation. Consequently $\bar{x}_\mathrm{l} \gg \bar{x}_\mathrm{b}$.

SUPPLEMENTARY REFERENCES

---

[1] Angelova, M. I. & Dimitrov, D. Liposome electroformation. *Faraday Discuss. Chem. Soc.* **81**, 303–311 (1989).

[2] Angelova, M. I., Soléau, S., Méléard, P., Faucon, J.-F. & Bothorel, P. Preparation of giant vesicles by external ac fields. kinetics and applications. *Pro. Colloid Polym. Sci.* **89**, 127–131 (1992).

[3] Estes, D. J. & Mayer, M. Electroformation of giant liposomes from spin-coated films of lipids. *Colloids Surf., B* **42**, 115–123 (2005).

[4] Mergny, J.-L. & Lacroix, L. Analysis of thermal melting curves. *Oligonucleotides* **13**, 515–537 (2003).

[5] Choi, K.-m., Kwon, I. C. & Ahn, H. J. Self-assembled amphiphilic DNA-cholesterol/DNA-peptide hybrid duplexes with liposome-like structure for doxorubicin delivery. *Biomaterials* **34**, 4183–4190 (2013).

[6] Ramachandran, A., Anderson, T. H., Leal, L. G. & Israelachvili, J. N. Adhesive interactions between vesicles in the strong adhesion limit. *Langmuir* **27**, 59–73 (2010).

[7] Mognetti, B. M., Leunissen, M. E. & Frenkel, D. Controlling the temperature sensitivity of DNA-mediated colloidal interactions through competing linkages. *Soft Matter* **8**, 2213–2221 (2012).

[8] Di Michele, L. *et al.* Effect of inert tails on the thermodynamics of DNA hybridization. *J. Am. Chem. Soc.* **136**, 6538–6541 (2014).

[9] Stan, C. A., Tang, S. K. Y. & Whitesides, G. M. Independent control of drop size and velocity in microfluidic flow-focusing generators using variable temperature and flow rate. *Anal. Chem.* **81**, 2399–2402 (2009).

[10] Pontani, L.-L., Jorjadze, I., Viasnoff, V. & Brujic, J. Biomimetic emulsions reveal the effect of mechanical forces on cell–cell adhesion. *Proc. Natl. Acad. Sci. U.S.A.* **109**, 9839–9844 (2012).
